# A Comparison of the Seasonal Movements of Tiger Sharks and Green Turtles Provides Insight into Their Predator-Prey Relationship

**DOI:** 10.1371/journal.pone.0051927

**Published:** 2012-12-19

**Authors:** Richard Fitzpatrick, Michele Thums, Ian Bell, Mark G. Meekan, John D. Stevens, Adam Barnett

**Affiliations:** 1 School of Marine and Tropical Biology, James Cook University, Cairns, Queensland, Australia; 2 Reef Channel, Cairns, Queensland, Australia; 3 School of Environmental Systems Engineering and the University of Western Australia Oceans Institute, The University of Western Australia, Crawley, Western Australia, Australia; 4 Australian Institute of Marine Science, c/o University of Western Australia Oceans Institute, Crawley, Western Australia, Australia; 5 Queensland Department of Environment and Heritage Protection, Townsville Queensland, Australia; 6 CSIRO Marine and Atmospheric Research, Hobart, Tasmania, Australia; 7 School of Life and Environmental Sciences, Deakin University, Melbourne, Victoria, Australia; 8 Fisheries, Aquaculture and Coasts Centre, Institute for Marine and Antarctic Studies, University of Tasmania, Hobart, Tasmania, Australia; University of Wales Swansea, United Kingdom

## Abstract

During the reproductive season, sea turtles use a restricted area in the vicinity of their nesting beaches, making them vulnerable to predation. At Raine Island (Australia), the highest density green turtle *Chelonia mydas* rookery in the world, tiger sharks *Galeocerdo cuvier* have been observed to feed on green turtles, and it has been suggested that they may specialise on such air-breathing prey. However there is little information with which to examine this hypothesis. We compared the spatial and temporal components of movement behaviour of these two potentially interacting species in order to provide insight into the predator-prey relationship. Specifically, we tested the hypothesis that tiger shark movements are more concentrated at Raine Island during the green turtle nesting season than outside the turtle nesting season when turtles are not concentrated at Raine Island. Turtles showed area-restricted search behaviour around Raine Island for ∼3–4 months during the nesting period (November–February). This was followed by direct movement (transit) to putative foraging grounds mostly in the Torres Straight where they switched to area-restricted search mode again, and remained resident for the remainder of the deployment (53–304 days). In contrast, tiger sharks displayed high spatial and temporal variation in movement behaviour which was not closely linked to the movement behaviour of green turtles or recognised turtle foraging grounds. On average, tiger sharks were concentrated around Raine Island throughout the year. While information on diet is required to determine whether tiger sharks are turtle specialists our results support the hypothesis that they target this predictable and plentiful prey during turtle nesting season, but they might not focus on this less predictable food source outside the nesting season.

## Introduction

Predators play important roles in ecosystems by influencing the distribution, behaviour and abundance of their prey and predator-prey interactions have long been recognised as important in ecosystem dynamics [1], [2], [3]. However predator-prey interactions can be difficult to observe and quantify. One way is to compare the spatial and temporal components of movement behaviour of potentially interacting species. By comparing movement patterns of predator and prey we can gain some understanding of when, where and how often predator and prey overlap spatially and therefore gauge the chance of interactions [4], [5], [6], [7]. Such studies can vastly improve our understanding of large mobile predators’ spatial use in relation to their prey and shed light on their foraging strategies. For instance, is the predators’ foraging strategy to move directly between distinct habitats exploiting seasonally abundant prey, or do they concentrate on targeting a specific prey and therefore their movements are dictated by the prey’s movements?

The tiger shark, *Galeocerdo cuvier,* is the apex predator in tropical coral reef systems, yet we know relatively little about the ecosystem role of this species in these environments. The limited information available suggests that they utilise large (hundreds of km) home ranges, within which they appear to move continuously between distant foraging areas [8], [9], [10], [11]. They can return to specific locations to take advantage of seasonally abundant prey like fledging albatross, *Phoebastria* spp. [11], [12]. Studies in some of these locations suggest that they have both demographic and behavioural effects on prey species [13], [14].

Although the diet of tiger sharks is broad, there is some evidence to suggest that they specialise on hunting air-breathing animals, particularly turtles [15], [16], [17] and they are considered the biggest predation threat (excluding humans) to these marine reptiles [18].

In the northern Great Barrier Reef, Australia, green turtles *Chelonia mydas* returning to natal rookeries during the austral summer are an abundant and predictable food source for tiger sharks. Raine Island and the surrounding sand cays in this location host the largest green turtle breeding population in the world [19] with turtle arrivals for nesting reaching a peak during the first week of December when an average of 5000 (range  = 250–12000/night) turtles can attempt to nest in a single night [20]. These animals exhibit strong site-attachment to Raine Island for up to four months during reproduction (courtship, copulation and nesting), exposing them to the possibility of concentrated predation pressure from tiger sharks for an extended period [19]. Tiger sharks are frequently observed scavenging on floating turtle carcases and occasionally seen attacking live turtles [19] (pers. obs.). Outside the reproductive season, green turtles disperse widely to distant foraging grounds [19], however the movements of tiger sharks in this region are unknown.

The coincidence of tiger sharks and this abundant food source of green turtles offered a unique opportunity to investigate the spatial relationship between this apex predator and its prey. We deployed satellite tags on tiger sharks and green turtles and analysed the movement behaviour of both species in order to: 1) determine whether tiger sharks concentrate their movements around Raine Island during the green turtle nesting season and 2) investigate the movement behaviour of green turtles and tiger sharks outside of the nesting season when turtles are more highly dispersed and not concentrated at Raine Island. We hypothesised that if tiger sharks were using the Raine Island area to focus their foraging effort on turtles (alive and dead) then their seasonal use of the waters near Raine Island should be highest during turtle nesting season and their movements might be less concentrated at Raine Island at the end of the nesting season when green turtles disperse widely to foraging grounds.

## Methods

### Ethics Statement

Research was approved and conducted under Australian Fisheries Management Authority Scientific Permit #901193 and Great Barrier Reef Marine Park Authority G11/33231.1.

Raine Island (11° 35′ S, 144° 02′ E) lies ∼80 km offshore from mainland Australia in the Far Northern Section of the Great Barrier Reef Marine Park. An elongate sand cay, approximately 830 m long and 430 m wide is located at the leeward end of an oval patch reef that is 3.5 km long and 0.75 km wide, with an area of 210 ha. The fringing reef of Raine Island slopes precipitously to meet the sea floor at depths of 200–300 m [19].

We deployed eight satellite-linked transmitters on green turtles and ten on tiger sharks at Raine Island in late spring and summer during the turtle nesting season between 2002 and 2007 (Table 1). The green turtles were all instrumented with SPOT transmitters (Wildlife computers, Redmond, Washington, USA). Shark deployments included one ST18 (Telonics, Mesa, Arizona, USA), four SPOT tags and five SPLASH tags (Wildlife computers, Redmond, Washington, USA). All tags relay position-only information via the ARGOS satellite network, except the SPLASH tags which also relay summaries of time at depth (±0.5 m) and time at temperature (±0.05°C) binned within 14 user defined data ranges over 6 h collection periods. One shark was tagged in consecutive summers. This individual was first tagged on 26^th^ November 2006 with SPLASH tag 72587, and then was recaptured 1 year later on 26^th^ November 2007. The SPLASH tag was removed and replaced with SPLASH tag 79975. The turtle tags were programmed to transmit from 04∶00–11∶00 h every day for December, January, February, March and April and the remaining months were duty cycled to transmit every 3 days. The rationale in selecting the transmit h was based on green turtles at Raine Island appearing to spend more time at the surface in the morning than in the afternoon when the wind speed increases (Bell pers. obs.). Therefore there would be a higher likelihood of obtaining uplinks from the tagged turtles basking in the calmest part of the day. The tiger sharks tags were programmed to transmit at all times.

**Table 1:** Details of satellite tag deployments on tiger sharks *Galeocerdo cuvier* and green turtles *Chelonia mydas* at Raine Island.

Species	Tag model	ID	Date tagged	Sex	Length (cm)	Deployment length	Locations d^−1^ (mean ± sd)
*G. cuvier* *	Telonics ST18	29222	21/2/2002	F	320	86	0.80±1.17
*G. cuvier*	SPOT 4	54738	23/11/2004	F	350	16	1.12±1.05
*G. cuvier*	SPOT 5	62849	18/12/2005	M	288	356	0.79±1.42
*G. cuvier*	SPOT 4	54739	3/12/2006	F	330	42	0.64±1.28
*G. cuvier*	SPOT 5	62848	8/12/2006	F	295	60	0.82±1.25
*G. cuvier*	SPLASH	79974	10/12/2007	M	292	209	0.68±1.05
*G. cuvier*	SPLASH	79973	13/12/2007	F	300	20	0.95±1.24
*G. cuvier*	SPLASH	79972	16/12/2007	F	296	231	0.42±0.93
*G. cuvier* **	SPLASH	72587	26/11/2006	F	350	42	3.35±2.11
	SPLASH	79975	26/11/2007			119	1.03±1.85
*C. mydas*	SPOT 5	79970	8/12/2007	F	115	408	1.01±1.25
*C. mydas*	SPOT 5	79971	9/12/2007	F	105	89	1.28±1.31
*C. mydas*	SPOT 5	79976	10/12/2007	F	109	151	1.74±1.55
*C. mydas*	SPOT 5	79977	11/12/2007	F	106	122	1.75±1.22
*C. mydas*	SPOT 5	88365	14/11/08	F	104	201	5.72±3.47
*C. mydas*	SPOT 5	88366	14/11/08	F	110	199	3.33±2.47
*C. mydas*	SPOT 5	88367	14/11/08	F	102	287	3.67±3.14
*C. mydas*	SPOT 5	88368	14/11/08	F	117	155	5±3.20

Lengths of sharks are total length and turtles are curved carapace length. Deployment length refers to the number of days between the tagging date and the date of the last satellite position fix.

* Shark died when caught in a fishing net.

** Shark was tagged in two consecutive summers.

Tiger sharks were attracted to a small boat using tuna heads threaded onto a buoyant rope, so the bait was floating on the surface and the rope attached to the boat. Once the shark had taken the bait, the boat was pulled along the bait line so that the boat was drawn close to the shark. A custom-designed tail clamp was then attached to the caudal peduncle with the use of a detachable 4 m pole. The clamp held a 5 m rope and large buoy, so once the clamp was attached it limited the swimming movement of the shark due to the drag of the buoy. The shark was then restrained using a harness at the back deck of the larger mothership vessel. The satellite transmitter was attached to the dorsal fin by two 5 mm diameter short, threaded nylon rods that passed through the fin and were secured on the other side by two washers and nuts, or a plastic plate and nuts [11]. The position of the transmitter on the fin was such that the antenna extended out of the water when the fin broke the surface. Transmitters were attached to green turtles that had successfully nested. The transmitter attachment procedure commenced immediately following oviposition or as the turtle was heading back to the sea. The SPOT tag was attached to the carapace using a fast drying epoxy resin (International Epiglass HT9000 Fast laminating resin). To avoid generating to much heat, we used less catalyst than the manufacturer’s instructions, so turtles were held in an enclosure for approximately 6 h allowing the epoxy sufficient time to fully set.

### Data Analysis

#### Movement behavior

The Bayesian state-space switching model (SSSM) developed by Jonsen et al. [21], [22] was applied to each individual tiger shark and green turtle track. Satellite-derived locations using the Service Argos telemetry system are reported at irregular time intervals and can be prone to considerable error [23]. The SSSM allows for regular position estimates to be inferred from the Argos satellite positions by taking into account error from the Argos location class (B, A, 0, 1, 2, 3) and the dynamics of the animal’s movement; the mean turning angle and autocorrelation in speed and direction [24]. It also identifies two discrete behavioural modes from these data, nominally, transiting and area-restricted search (ARS) behaviour, assuming that while transiting, turn angles should be closer to 0 and autocorrelation should be higher than when in ARS [25]. While area-restricted search behaviour is associated with foraging [26] it can also be resting or breeding behaviour [27], [28].

The SSSMs were fitted using the freely available software, JAGS 3.2.0 [29] from R: A Language and Environment for Statistical Computing [30] using code developed by Ian Jonsen (bsam) [21], [22]. We ran two Monte-Carlo Markov Chains (MCMC) for each model with 60 000 iterations following a 30 000 burn-in (thin  = 10). We used a 12 h time step for tiger sharks and 6–12 h time step for the turtles depending on the temporal resolution of the ARGOS data (Table 1). The SSSM classifies behaviour by using the means of the MCMC samples and delineating a cut-off at 1.5. Mean estimates below 1.5 were considered to represent transiting and estimates above 1.5 were considered to represent area-restricted search [24] and mean estimates of 1.5 were considered uncertain.

To assess whether shark movement was more concentrated at Raine Island during the green turtle nesting season we calculated the distance between each location and Raine Island for each shark using the sp package in R. We designated the period from November to February as the turtle nesting season based on the green turtle movement behaviour. We used linear mixed effects models to model the distance from Raine Island as a function of turtle season (nesting or not nesting). The random effect was the individual shark and we used the corAR1 function to account for the within-group correlation structure. We log-transformed the response to normalise its distribution and fitted the models in R using package nlme [31]. We used an information theoretic approach [32] to test the hypothesis by comparing the weight of Akaike’s information criterion corrected for small sample sizes (*w*AIC*_c_*) of the slope model (Distance from Raine ∼ season) to the intercept-only model (Distance to Raine ∼1) [32].

To determine whether sharks showed variation in diving behaviour or thermal environments used between behavioural modes, we calculated the proportion of observations within each depth and temperature bin for each behavioural mode for each animal with diving data. All means presented in the text are accompanied by the standard deviation.

## Results

The number of locations obtained per day was 0.99±0.82 for tiger sharks and 2.94±1.77 for green turtles (Table 1). For sharks, there were periods within each track with no recorded satellite locations. This resulted in the low mean number of positions calculated for each shark (Table 1). The median length of these periods ranged from 1–4 days. The deployments provided data for 16–365 days for the sharks and 89–408 days for turtles (Table 1). There was little uncertainty in the behavioural mode estimates with only 0.3% of state estimates at 1.5. The largest proportion of time was spent in area-restricted search mode for both turtles (0.95±0.03) and sharks (0.90±0.12).

**Figure 1:**
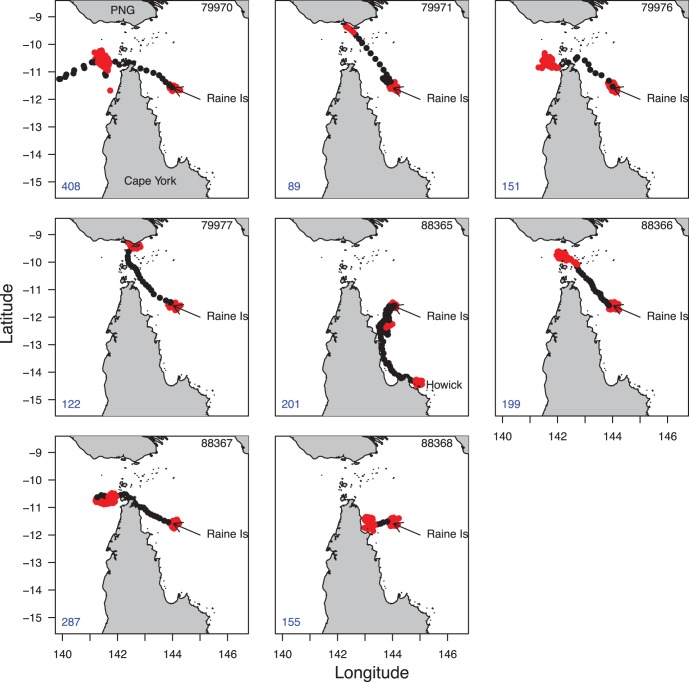
Movement path of each green turtle. Turtle ID is indicated in the top right and duration of the deployment in the bottom left of each map. Each turtle’s path is coded by behavioural mode; red = area-restricted search and black = transit. Maps show Cape York Peninsula, Raine Island and the bottom of Papua New Guinea (PNG) (top).

From the deployment date, green turtles spent 76±24 days in area-restricted search mode around Raine Island (Fig. 1). Apart from one turtle (88365) that switched to transit mode in early December, the turtles switched to transit mode from early February to mid March (Julian day range: 33–74), equating to a mean Julian day of 51±18 (20^th^ February). The switch to transit mode was accompanied with a largely northward migration (Fig. 1). This switch probably relates to the transition between the nesting phase and the post-nesting migration to foraging grounds. Turtles spent 9.16±7.20 days in transit mode before a long period in area-restricted search mode (putative foraging) which lasted for the remainder of their deployments (Table 1) predominantly in the Torres Strait (Fig. 1). One turtle headed directly west of Raine to forage off the coast of Cape York Peninsula and another went south to the Howick group (Fig. 1). When in the nesting phase (based on the time each turtle switched to transit mode) around Raine Island, turtles were 5.97±2.02 km from Raine Island. On their foraging grounds they were 272.03±72.51 km from Raine Island.

**Figure 2:**
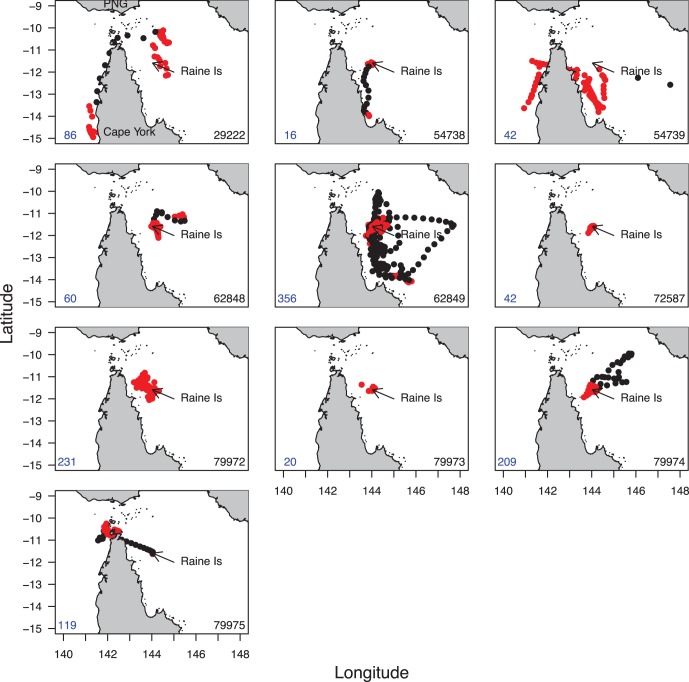
Movement path of each tiger shark. Shark ID is indicated in the top right and duration of the deployment in the bottom left of each map. Each shark’s path is coded by behavioural mode; red = area-restricted search and black = transit. Maps show Cape York Peninsula, Raine Island and the bottom of Papua New Guniea (PNG) (top).

Tiger shark movement patterns were more variable than those of green turtles with approximately half the animals showing discrete periods of area-restricted search separated by transit movement (Fig. 2). Sharks mainly displayed concentrated area-restricted search movements when they were in the vicinity of Raine Island. Sharks did not show concentrated area-restricted search in any other discrete location, except for shark 79975. This shark left Raine Island three days after tagging, spending 12 days in transit to the Torres Strait Islands (Fig. 2), in a similar region to that of green turtle foraging areas where it proceeded to spend the rest of the time (105 days). These results are likely, in part, to be related to the shorter (on average) deployment lengths for sharks than turtles.

**Figure 3:**
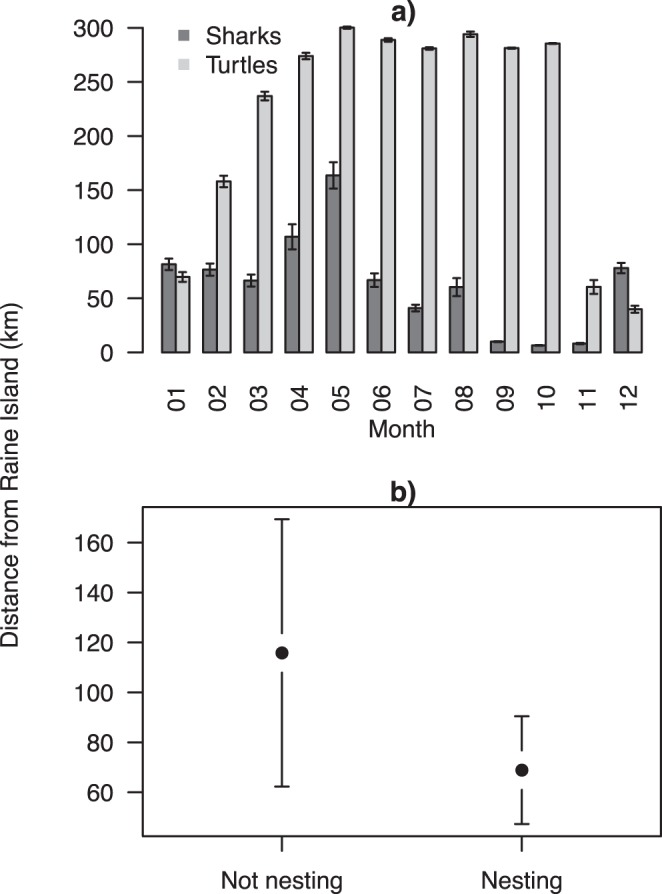
Mean distance from Raine Island for tiger sharks and green turtles. Mean and standard error distance from Raine Island for each month for all tiger sharks and green turtles (a). Mean and standard error distance from Raine Island for tiger sharks during the green turtle nesting season (nesting) and outside the nesting season (not nesting) (b).

**Figure 4:**
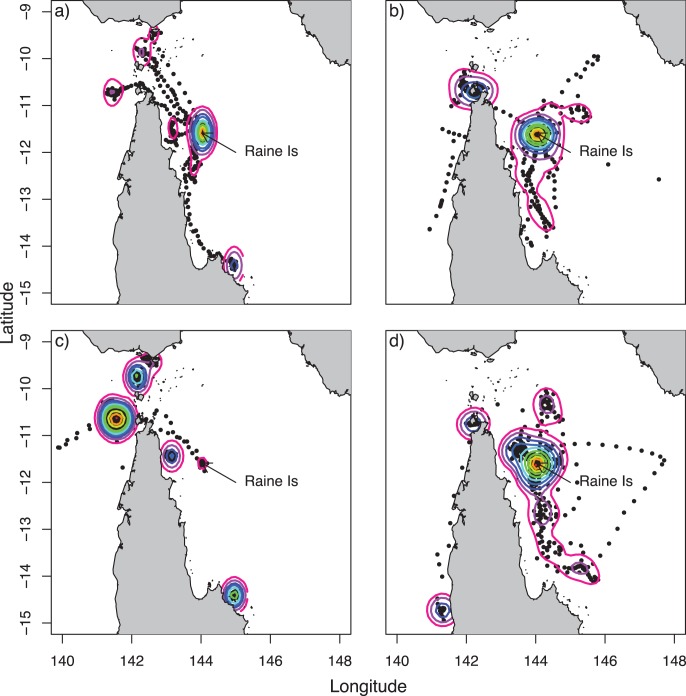
Kernel density of green turtle and tiger shark positions with reference to green turtle nesting season. Plots show green turtles (a) and tiger sharks (b) during the green turtle nesting season and green turtles (c) and tiger sharks (d) outside the nesting season. Black dots show shark locations. Warmer colours correspond to more points. Maps show Cape York Peninsula and Papua New Guinnea at the top left and right.

**Figure 5:**
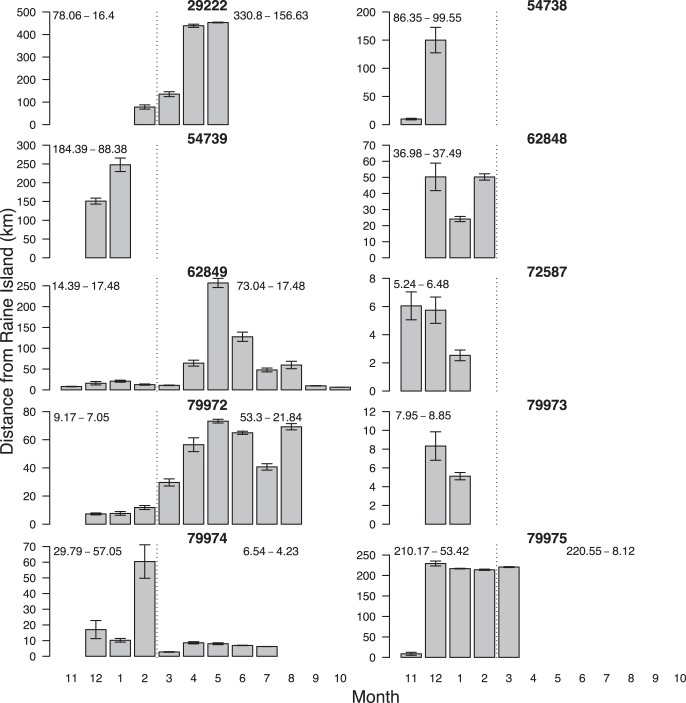
Monthly distance from Raine Island for individual tiger sharks. Plots show the mean and standard error of monthly distance from Raine Island for each tiger shark. Dashed line shows the nesting period for which turtles were in area-restricted search mode around Raine Island prior to the switch to transit movement mode and migration to the foraging grounds. Numbers to the left of the dashed line refer to the mean and sd of distance from Raine Island during the green turtle nesting period and numbers to the right of the dashed line shows the mean and sd of distance from Raine Island outside the green turtle nesting period. Note that the y-axis is different for each shark to allow comparisons between months for each shark.

Tiger sharks largely remained in the vicinity of Raine Island (Fig. 3a). During the green turtle nesting phase (Nov – Feb) the sharks were 68.89±71.58 km from Raine Island and outside of the nesting phase they were 115.82±131.08 km from Raine (Fig. 3b). We found no evidence to suggest that shark distance from Raine Island was different in the nesting season compared to outside the nesting season with the intercept only model (null model) having much higher support (*w*AIC*_c_*  = 0.78) than the slope model (*w*AIC*_c_*  = 0.22) (Figs. 3 and 4). However, some sharks did travel further afield. Sharks 79974 and 62849 moved into the Coral Sea, to the south-east of Papua New Guinea (Figs. 2 and 5). These sharks also had two of the three longest data records (209 and 356 days) (Table 1). Interestingly, these transits were of short duration and didn’t result in area-restricted search behaviour in the Coral Sea and both sharks returned to Raine Island outside of the green turtle nesting season (Fig. 5). In contrast, shark 79972 which had location data for 231 days stayed relatively close to Raine Island, never leaving the northern Great Barrier Reef region and never showing transit movement (Figs. 2 and 5). While there were no differences in proximity to Raine Island overall between nesting and non-nesting season, half of the shark deployments conformed with the prediction of being closer to Raine Island in the green turtle nesting season. For the tiger sharks with long enough deployments (62849, 79972 and 79974), two of these fit the prediction (Fig. 5). During the 2010/11 summer expeditions to Raine Island two satellite tagged tiger sharks were also observed feeding on a dead turtle, but neither animal was recaptured.

**Figure 6:**
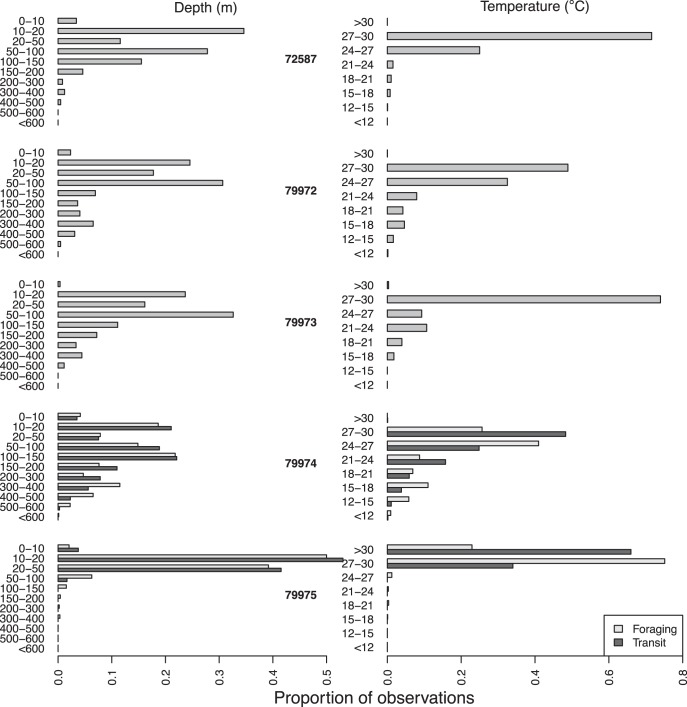
Time spent in each temperature and depth bin. Plots show the proportion of time spent in each depth (left plots) and temperature (right plots) bin for each behavioural mode for the tiger sharks with SPLASH tags. Note that the first three sharks did not have any transit behaviour.

The majority of tiger sharks with depth data spent most of their time in the 10, 20 and 50 m depth bins with smaller proportions of time in the depth bins up to 400 m (Fig. 6). One of the sharks that travelled into the Coral Sea (79974) spent more time in these deeper depth bins than the other sharks. A similar pattern was seen with time at temperature with the majority of sharks inhabiting temperatures around 27–33°C (Fig. 6). There was not a close correspondence with switches in one behavioural mode to the other and variation in time at depth and time at temperature (Fig. 6).

## Discussion

There is now growing recognition of the structuring role of top-order predators in ecosystems [33], [34] and there is a need for behavioural studies that integrate information on the movements and interactions of apex predators and their prey [7]. Our study is one of the few to investigate the movements of a top-order marine predator and its air-breathing prey over a large (10–100 s km) spatial scale (for studies at scales of m–km see [4], [14]). On average, tiger sharks spent most of their time in the vicinity of Raine Island, where the majority of area-restricted search behaviour took place, irrespective of the green turtle season. When green turtles left Raine for their foraging grounds in the Torres Strait, tiger sharks did not conduct migrations to other discrete foraging grounds. Our results suggest that while tiger sharks might target green turtles when they are concentrated at Raine Island during the nesting season, they might not focus principally on this prey source outside the nesting season when turtles are widely dispersed. These observations further support the view that tiger sharks are generalist feeders [35].

The tracks of tiger sharks were highly variable both spatially and temporally. The movement further afield by some tiger sharks in autumn occurred after the green turtles had migrated away from Raine Island in March. Some sharks (e.g. 54738, 54739 and 79975) had already moved over 150 km away from Raine Island prior to green turtles leaving and others were present at Raine Island and the surrounding areas outside of the peak green turtle nesting season. Although sharks 62849 and 79974 moved large distances away from Raine Island, they both returned and spent time at the island outside of the green turtle nesting season. The return of these sharks to Raine Island was not simply a consequence of their relatively long data records (356 and 209 d respectively), since the shark with the 2^nd^ longest data record (79972) spent 231 days in the Raine Island region and showed no transit movement. Such individual variability in movement behaviour and habitat use among individual tiger sharks appears typical of the species, since tagging studies in other localities have recorded similar patterns [9], [11], [36]. For example, at the French Frigate Shoals, tagging showed that some animals were present year round, whereas others visited the atoll in summer to forage on fledging albatross and then departed before returning in subsequent years [11]. In Shark Bay, Western Australia tiger shark numbers increase when dugongs are abundant, but as with the turtles in the current study the long-term movements of tiger sharks do not match those of dugongs [9]. Tiger sharks tagged in Shark Bay showed variable movement patterns outside the seasonal occurrence of their prey with some individuals remaining in the Shark Bay region while others made larger excursions that included offshore waters [9]. Meyer et al. [10], [11] suggested that tiger shark movements presumably include some element of exploration, enabling them to discover new foraging locations and over time, build up detailed spatio-temporal maps of productive prey patches. Individual differences in movement behaviour and the use of key habitats have also been observed in broadnose sevengill sharks, *Notorynchus cepedianus* [37], [38], a species that fills an apex predator role in temperate coastal waters.

With the exception of shark 79975 that moved to the north of Cape York, a similar area that four tagged green turtles also migrated to, none of the tiger sharks moved to green turtle foraging grounds to the north of Raine Island. These results imply that either there is sufficient other types of prey in the region of Raine island or that other species of turtles are present in the area to provide food for the sharks. We would not expect that some green turtles remain to forage as the area around Raine Island is relatively deep with no known seagrass habitat and the closest recognised foraging grounds are ∼80 km away in the adjacent coastal region [19], [20]. While there is seagrass habitat in the coastal region adjacent to Raine Island, the movement behaviour and the kernel density plots showed that tiger sharks still concentrated their movements at Raine Island both inside and outside the turtle nesting season. Even if turtles are present at Raine Island outside of the nesting season, they are much more widely dispersed at this time making this food source less predictable and searching over larger areas would be required for tiger sharks to continue to target them. Multi-season data are required to conclusively answer the question, specifically to determine whether tiger sharks arrive at Raine Island at the start of the nesting season. Our tagging subjects were all caught at Raine Island and the deployments therefore commenced during green turtle nesting season. In addition, we had a low sample size with which to examine the hypothesis as only half the sharks provided data outside the green turtle nesting season. However, our results suggest that movement of this predator and its turtle prey are not strongly linked throughout the entire year.

Three tiger sharks made forays into the open ocean beyond the edge of the continental shelf. These excursions only lasted between 6–8 days. Similarly, tiger sharks in Shark Bay, Western Australia moved offshore into waters with depths of ∼800 m, but did not remain there for protracted periods [9]. Other tagging studies in Hawaii and French Frigate Shoals recorded regular offshore movements of tiger sharks across deep waters [8], [11]. Tiger sharks tagged at Raine Island spent most of their time above 100 m but made dives exceeding 600 m depth. This is very similar to the depth behaviour exhibited by tiger sharks at the French Frigate Shoals [11]. The reasons for the offshore movements in our study are unknown; clearly they had little to do with adult green turtles, since these animals migrated through shelf waters. It is possible that these movements are exploratory since they displayed transit type movements of short durations. Offshore movements of some tiger sharks in Hawaii were linked to patterns of oceanic productivity [11] and there are anecdotal reports of seasonal bursts of productivity in the Coral Sea that are related to spawning events of tunas and myctophid fishes.

### Tag Performance on Sharks

The tiger sharks tracked for 209, 231 and 356 days are the longest satellite tag deployments on this species (but see [36] for a track of 297 d) and with the exception of salmon sharks, *Lamna ditropis* (*n*  = 68, 6–1335 days), they are also some of the longest satellite tracks for any shark [39]. However, the remaining deployment periods (16–119 days) were comparable to results of previous studies that attached satellite tags to dorsal fins of tiger sharks [9], [11], [36]. Suspected reasons for the premature cessation of data uplinks are the exhaustion of batteries, antenna breakage, animal mortality, damage to the tag, detachment of tags from the animals, and the biofouling of the saltwater switch, which may be particularly problematic in tropical waters [11], [40]. The antenna of the tag recovered in our study was covered in algae causing the antenna to bend (Fig. S1). However, the cessation of data uplinks after 42 days was probably due to battery failure as the level of fouling to bend the antenna could not occur in such a short time. Tags deployed on tiger sharks transmitted on average approximately one location per day. However, the raw locations were not regularly spaced through time with high daily variation and gaps in the data record. Even though the tiger shark tracks had periods with fewer raw satellite locations than the 12 h interval at which the SSSM locations were being estimated we do not think that the gaps were large enough to impact the accuracy of the SSSM location estimates, see [28]. This is quite common for tracking studies on marine vertebrates, due to limited and/or short duration surface intervals, biofouling or tag defects [40]. These problems influence the number of location fixes and suggests that Argos satellite tags may only provide limited movement information for this species. The newer models of satellite tags with Fastloc™ GPS are capable of acquiring the data required for a location fix in a much shorter period of time and with greater location accuracy. These tags will be beneficial in future work as short surface intervals will still result in high quality location data [41]. Also, combining satellite tag technology with other methods such as acoustic tracking and/or stable isotopes should provide more comprehensive information on movement patterns, habitat use and species interactions [11], [37], [38], [42]. Approaches such as the SSSM used here are therefore essential in order to make the most out of the typically low spatial and temporal resolution data obtained from Argos tracking studies on marine vertebrates.

### Conclusion

Individual tiger sharks displayed high spatial and temporal variation in movement behaviour which was not closely linked to the movement behaviour of green turtles. On average tiger shark movements were concentrated at Raine Island throughout the year. The concentrated spatial and temporal overlap of tiger sharks and green turtles during the green turtle nesting season on Raine Island suggest these predators could have a significant effect on green turtle behaviour and populations during the nesting season. Given that green turtles (and sea turtles generally) are widely dispersed outside the nesting season and that tiger sharks remained largely concentrated at Raine Island year round, we suggest that tiger sharks do not focus on this less predictable food source outside the green turtle nesting season. While our approach was based solely on movement data and cannot conclusively determine whether tiger sharks target green turtles, it has provided information on the spatial and temporal overlap between these two species. Closer examination of the predator-prey relationship between these two species requires spatial data at finer scales over longer time scales combined with information on tiger shark diet. Our approach has resulted in more accurate tracks, revealed changes in behaviour and provided insight into the predator-prey relationship between tiger sharks and green turtles.

## Supporting Information

Figure S1 **Satellite transmitter recovered from tiger shark.** Picture shows algal growth on the recovered transmitter (A). Satellite transmitter still attached to shark showing how the algal growth bends the antenna (B).
(TIFF)
