# Supplementary figures and images for: A Comparison of the Seasonal Movements of Tiger Sharks and Green Turtles Provides Insight into Their Predator-Prey Relationship
Source: PLoS One. 2012 Dec 19;7(12):e51927. doi: 10.1371/journal.pone.0051927 (PMC3526478; doi:10.1371/journal.pone.0051927)

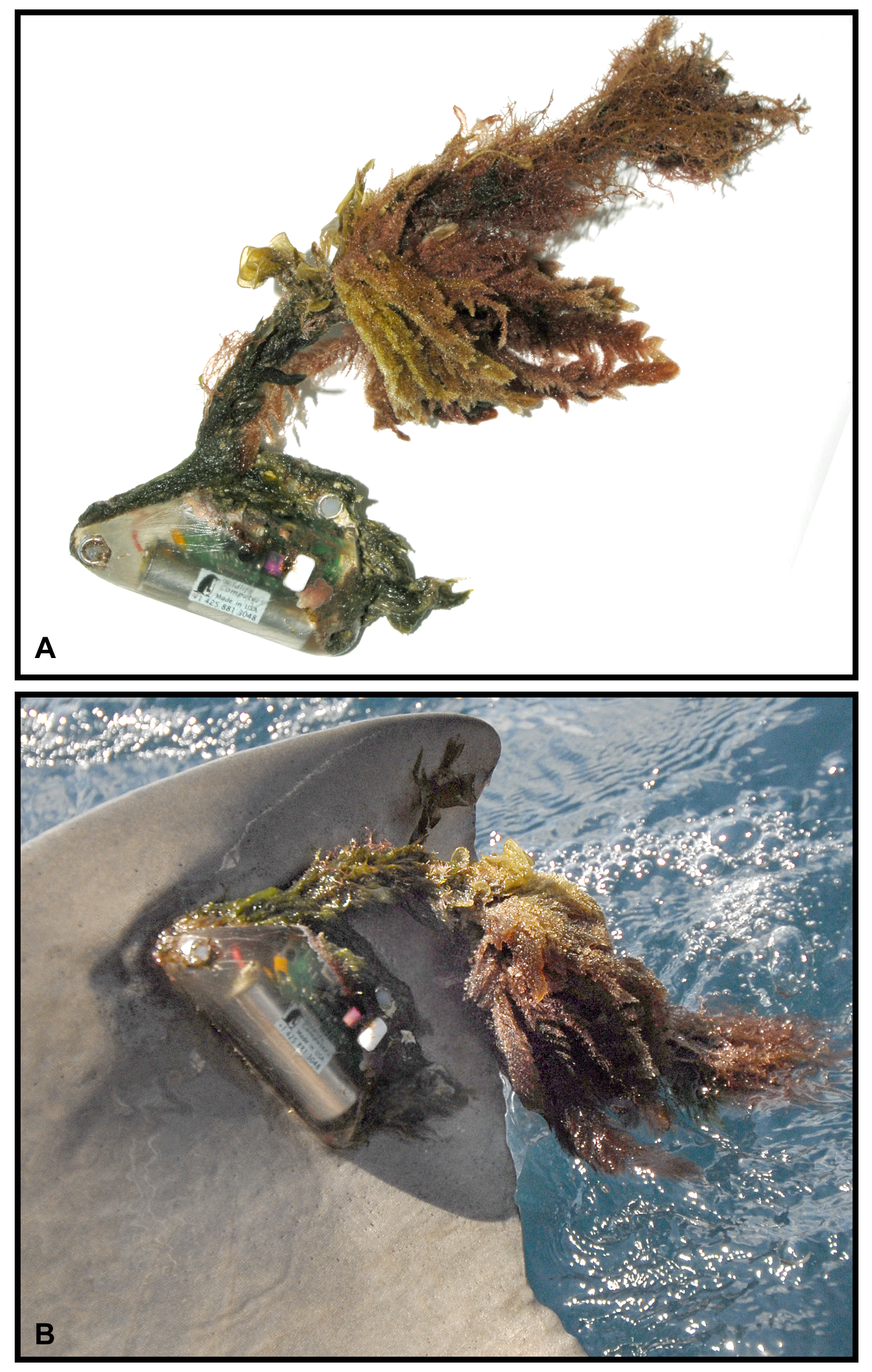

Supplement: Figure S1 — Satellite transmitter recovered from tiger shark. Picture shows algal growth on the recovered transmitter (A). Satellite transmitter still attached to shark showing how the algal growth bends the antenna (B). (TIFF) [file pone.0051927.s001.tiff]
